# Understanding low colorectal cancer screening uptake in South Asian faith communities in England – a qualitative study

**DOI:** 10.1186/s12889-015-2334-9

**Published:** 2015-10-01

**Authors:** Cecily K. Palmer, Mary C. Thomas, Lesley M. McGregor, Christian von Wagner, Rosalind Raine

**Affiliations:** Department of Applied Health Research, University College London, 1-19 Torrington Place, London, UK; Department of Epidemiology and Public Health, Cancer Research UK Health Behaviour Research Centre, University College London, 1-19 Torrington Place, London, UK

**Keywords:** South Asian, Minority ethnic groups, Colorectal cancer screening, Bowel cancer screening, Guaiac faecal occult blood test (gFOBt), Access, Uptake, Health services, Qualitative, Key informant

## Abstract

**Background:**

Colorectal cancer screening uptake within the South Asian population in England is approximately half that of the general population (33 % vs 61 %), and varies by Muslim (31.9 %), Sikh (34.6 %) and Hindu (43.7 %) faith background. This study sought to explore reasons for low uptake of CRC screening in South Asian communities and for the variability of low uptake between three faith communities; and to identify strategies by which uptake might be improved.

**Methods:**

We interviewed 16 ‘key informants’ representing communities from the three largest South Asian faith backgrounds (Islam, Hinduism and Sikhism) in London, England.

**Results:**

Reasons for low colorectal cancer screening uptake were overwhelmingly shared across South Asian faith groups. These were: limitations posed by written English; limitations posed by any written language; reliance on younger family members; low awareness of colorectal cancer and screening; and difficulties associated with faeces. Non-written information delivered verbally and interactively within faith or community settings was preferred across faith communities.

**Conclusions:**

Efforts to increase accessibility to colorectal cancer screening in South Asian communities should use local language broadcasts on ethnic media and face-to-face approaches within community and faith settings to increase awareness of colorectal cancer and screening, and address challenges posed by written materials.

## Background

Colorectal cancer (CRC) is the third most common cancer in the UK [1]. Regular screening using the guaiac faecal occult blood test (gFOBt) can reduce the risk of dying from the disease by 16 % [2, 3]. Established in 2006, the English NHS Bowel Cancer Screening Programme (BCSP) invites adults aged 60–74 who are registered with a GP to participate in screening for CRC every two years. Eligible adults are sent invitation materials and a gFOBt kit by post. To participate in screening individuals must smear two small samples from three separate bowel movements on to the gFOBt kit. The kit is returned to a laboratory in a special envelope, where the samples are tested for traces of blood [4].

Overall uptake within the BCSP is 54 % [5] but this masks much lower rates in South Asian communities which vary from 31.9 % in the Muslim community, 34.6 % in the Sikh community and 43.7 % in the Hindu community [6–8]. South Asian minority ethnic communities (comprising Indian, Pakistani and Bangladeshi groups) make up 7.5 % of the population of England and Wales [9, 10]. Low uptake of CRC screening in the UK has continued to be identified in areas with higher ethnic diversity [5, 11, 12], and within all South Asian religo-linguistic groups even when age, deprivation (defined as area-based deprivation calculated using census data) and gender are adjusted for [8, 13]. Uptake across screening programmes has consistently been lower in London than the rest of the country, attributed, in part, to its diverse population [14–16].

Research exploring acceptability of CRC screening (by gFOBt) among UK minority ethnic communities is limited to a single study undertaken prior to the initiation of the BCSP which involved focus group discussions with participants from diverse minority ethnic groups, most of whom had not yet been invited to CRC screening [7]. The study found that participants supported the principle of screening and gFOBt completion once it had been explained to them. However, many participants would not respond to postal invitations without prior warning being given, preferably by trusted local sources; invitees would require support from family members to translate information materials; and translated materials could pose problems due to the dialects spoken and/or poor literacy in their first language [7]. Importantly, no studies have explored explanations for the differences in gFOBt uptake identified in the Muslim, Sikh and Hindu faith communities [7]. The UK South Asian community comprises multiple ethnic groups with diverse religious, linguistic, cultural and geographical origins, and there have been calls for greater attentiveness to this heterogeneity in health research involving participants of South Asian origin [17, 18].

Using a key informant approach, our study aimed to identify reasons for low uptake of CRC screening in South Asian communities, and to explore reasons for the differences in CRC screening uptake between Muslim, Sikh and Hindu faith groups. We also aimed to identify possible methods by which uptake might be improved.

## Methods

### Recruitment and sampling

Key informant interviews have been used across a number of social research disciplines to generate contextually detailed and culturally informed knowledge about community members’ use (or non-use) of health services [19, 20]. We therefore aimed to recruit key informants from the three largest South Asian faith communities (Islam, Hinduism and Sikhism) to generate insights regarding acceptability and accessibility of an invitation to the BCSP.

Approximately half of the UK South Asian minority ethnic population live in London [10, 21]. We recruited key informants via direct approaches to 26 London-based faith and community organisations that provide services specifically to South Asian communities. Organisations were identified via internet searches and professional and personal contacts. A snowball technique was also used to generate further contacts for possible participation. Potential participants at each organisation were contacted by email or telephone, provided with information about the study and invited to participate. Key informants were purposively identified to ensure that they held an embedded role within an organisation serving one of the three main South Asian faith communities (Islam, Hinduism and Sikhism), and that they spoke English at a level suitable for interview. Organisations continued to be identified and approached until an equal number of informants had been recruited for each faith community. All willing informants were interviewed and none were turned away. In addition, we recruited General Practitioners (GPs) working in areas with large South Asian populations via two Comprehensive Local Research Networks (North West London and East London), and personal contacts.

In total 16 key informants were recruited to the study. Twelve held roles across a total of ten community or faith organisations; four were recruited from Gurdwaras or social groups (Sikh community), four were recruited from Mandirs or social groups (Hindu community), and four were recruited from community groups providing services for the Muslim community in East London whose members mostly originate from the Sylhet region of Bangladesh. The final four informants were general practitioners serving areas with a large South Asian minority ethnic population in the London boroughs of Barnet, Tower Hamlets, Harrow and Redbridge (see Table 1).

**Table 1:** Key informant participant sample

Interview number	Organisation key informant recruited from	Area	South Asian community represented	Sex
1	Social/Community group linked to Gurdwara	Southall, West London	Sikh community	M + F
2	Mandir	Neasden, North West London	Hindu community	F
3	Community health charity	Tower Hamlets, East London	Muslim community (Bangladeshi)	F
5	Community centre	Tower Hamlets, East London	Muslim community (Bangladeshi)	M
6	Community charity	Tower Hamlets, East London	Muslim community (Bangladeshi)	F
7	Mandir	Wembley, North West London	Hindu community	F
8	Community charity	Tower Hamlets, East London	Muslim community (Bangladeshi)	F
9	Gurdwara	Hounslow, West London	Sikh community	M
10	Social/Community group	Barnet, North London	Hindu community	M
11	Mandir	Southall, West London	Hindu community	M
12	Gurdwara	Ealing, West London	Sikh community	M
13	General Practitioner	Barnet, North London	Mixed with large Indian Gujarati speaking population	F
14	General Practitioner	Tower Hamlets, East London	Bangladeshi largely Sylheti speaking population	F
15	General Practitioner	Harrow, North West London	Mixed with large Indian Gujarati speaking population	F
16	General Practitioner	Redbridge, Essex	Mixed Pakistani, Indian and Bangladeshi population	M

### Data collection

Between May and December 2013, semi-structured face-to-face interviews were undertaken with 16 key informants. A topic guide was developed for use in each interview and included five main areas of interest; informants were asked to describe how people within the communities they represented might react when the BCSP invitation and test kit came through the post; how easy it would be for community members to take part in screening in its current form; why people might not take part in screening when invited; why uptake of CRC screening might be low in their community; and how uptake of screening might be improved within their community. Each informant was presented with a copy of the invitation and screening materials sent out by the BCSP, including the gFOBt kit, to add context to the questions asked and to encourage descriptive and detailed responses. All interviews were conducted in English and lasted between 25–40 min. A nominal donation was made to each community/faith organisation from which an informant was recruited in recognition of their contribution to the study.

### Analysis

An inductive analytical approach was used to generate themes from the data [22]. Each transcript was read and coded by at least two out of three authors (CP, MT, LM), and the data were initially analysed separately by faith community. Authors compared themes to identify commonalities and differences between data generated across the three faith communities.

### Ethics

Ethical approval for the study was granted by NRES Committee London-Bromley (reference 11/H0805/7). NHS R&D approvals were gained for interviews undertaken with GP informants. Written informed consent was obtained from all participants.

## Results

Key informants provided detailed commentaries about how their communities would be likely to respond to an invitation to the BCSP. We present five main themes relating to low uptake of CRC screening that were described across faith groups: limitations posed by written English; limitations posed by any written language; reliance on younger family members; low awareness of CRC and screening; and difficulties associated with faeces. We also report suggestions to increase accessibility and uptake of screening.

### Limitations posed by written English

Unanimously, key informants described how many South Asian elders eligible for CRC screening would not be able to engage with the letter and accompanying information that is sent by the BCSP, because they have limited ability to read and speak in English.

Some of the elderly […] don’t necessarily have English as their first language. (Hindu Community informant: 2)

…quite a lot of the older people don’t speak English very well. (Sikh Community informant: 9)

In recognition of the language needs of the population, the BCSP offers translated materials, which are available upon request by telephoning the helpline. However, calling the helpline was perceived to require considerable motivation, potentially in the absence of knowledge regarding what the recipient is seeking information about.

So what kind of person would ring [the BCSP helpline], it would be someone who is very motivated to want to do it. Or very interested in these letters coming through but they don’t understand it. (GP informant: 14)

Therefore, informants identified that variable proficiency in reading English would make accessing translated materials challenging, and would be likely to require a family member to mediate. Finally, even for those who do read English, informants perceived that the BCSP invitation materials comprised too much information.

It’s a lot to take in […] It’s just too much, to be honest. (Hindu Community informant: 2)

### Limitations posed by any written language

Communications using written words **in any language** were repeatedly described by informants as unappealing, and lacking impact and importance within their communities, and for these reasons informants suggested that postal communications were often overlooked. This lack of impact was vividly described in relation to the Bangladeshi Muslim faith community, the majority of whom speak Sylheti, which is rarely used in its written form, and whom were unlikely to be able to read in Bengali, meaning that written translations of screening information would be of no use.

They don’t function in a written way […] written information does not give people the ability to go and do what needs doing. (GP informant: 14)

It was noteworthy that across the faith communities, human interaction involving face-to-face discussion, verbal descriptions, and demonstrations were described as the favoured means of communication to effectively share information.

Our people are more visual learners […] they rather see and hear before they make any decision. (Muslim Community informant: 6)

Somebody who is like 60 or 65 […] they probably need some human touch where people can come and explain to them (Hindu Community informant: 11)

Informants recommended that these interactive approaches be undertaken within faith and other community settings. Approaching community members in a familiar place, and communally rather than individually, was endorsed as a means of increasing the understanding and confidence of community members, enabling them to more readily engage in screening.

I think it’s coming into the community […] at our Mandir it works really well because you’re capturing the audience in their home, as it were, and they feel comfortable […] as long as, of course, it’s in their language as well. (Hindu Community informant: 2)

### Reliance on younger family members

Informants across faith communities reported that it was common for sons and daughters to translate and interpret written materials for older members of the family, and that support of this kind would be required for the BCSP invitation materials. Informants suggested that participation in screening may therefore be heavily mediated by younger family members, who may further make their own judgments about the importance of the screening invitation.

She [my mum] said to me, there’s a letter for me from the doctor. I came and looked at it but I didn’t put that much emphasis on the importance, I didn’t encourage her to take up. (Muslim community informant: 3)

Informants also considered that community members would require help with collecting and sampling faeces to complete the gFOBt kit, but that the personal nature of the test would mean younger family members would be less likely to assist.

This is something very personal you know toilet is something you don’t dare - even dare to ask children you know ‘can you do that?’ (Hindu Community informant: 10)

### Low awareness of cancer and screening

Key informants reported low awareness of CRC and CRC screening within their communities, and suggested that participation in screening would increase if communities were given culturally accessible information about the purpose and value of screening, and the practical side of gFOBt kit completion.

Unless they understood how important it was, they wouldn’t do it. […] you would need to tell them what the facts and figures are, why it’s important for them to do it, what the risks are. (Muslim Community informant: 8)

Informants also identified low awareness of cancer being potentially curable and reported that cancer was perceived to be serious, frightening and final.

Cancer is one of those things that everyone regards as you can’t do anything about it, once you get it you get it, and that’s end of. (Muslim Community informant: 8)

Informants representing Sikh faith communities described a particular reluctance to disclose a cancer diagnosis or talk about cancer more generally which was linked to low awareness within the community. This reluctance was explained in terms of a social stigma surrounding cancer and fear of the potentially negative reactions from the wider community that may be elicited in response to cancer.

…within the family someone will get cancer and they don’t talk about it. It’s just a social stigma on things […] they think that ‘what will other people think?’ (Sikh Community informant: 9)

Informants suggested that due to low awareness of screening, the BCSP invitation was likely to be perceived as having come ‘out of the blue’, and that this perception would be further reinforced by screening invitations being sent from a national source, rather than from a familiar person or organisation (e.g. a GP). Informants proposed using non-written mediums such as Asian TV or radio, to raise awareness of the BCSP, and to prepare people for the forthcoming invitation and gFOBt kit.

I would get something on radio and that would get the message across and then at least then they’ll be looking out for the letters. (Sikh Community informant: 9)

Informants suggested that awareness-raising should include positive information about early-diagnosis and cancer curability to counteract some of the fear surrounding cancer, and recommended that information be provided face-to-face and ‘in language’ to groups within faith and community settings, incorporating gFOBt kit demonstrations, and opportunities to ask questions. Health fairs using these approaches were already taking place within some of the faith settings represented by informants.

### Difficulties associated with faeces

Key informants suggested that the requirement to complete the gFOBt kit with samples of faeces and to store the kit over a period of days would be considered unpleasant and compromising to hygiene for some community members.

Doing something like this and having it out for 3 days with faecal matter on it is totally abhorrent to them. (GP informant: 13)

Informants suggested that a simplified test that required a one-off sample might overcome some difficulties with test completion, and proposed that community members be given the option to take the gFOBt to their GP or practice nurse to seek explanation and practical instruction.

## Discussion

Our study has identified overwhelming commonality in the reasons for ongoing low uptake of CRC screening amongst the Hindu, Sikh and (Bangladeshi) Muslim communities represented. In common with previous research we identified that across faith communities, the delivery of CRC screening using a written approach directed at the individual was considered likely to be inaccessible to a significant number of South Asian people of screening age [7, 16, 23]. Communication via written materials came across as being particularly inappropriate for the East London Bangladeshi Muslim community due to the largely oral culture of this community. This finding may partially explain why screening uptake is particularly low in the South Asian Muslim community and warrants further exploration [7]. In common with previous research we identified reliance on younger family members to interpret and navigate health information and that consequently family members may make judgements on behalf of elders regarding the value of screening [7].

Our finding of low awareness of the existence and purpose of CRC screening across South Asian faith communities confirm previous findings across screening programmes [7, 15, 16, 23, 24]. A number of studies also found low knowledge and awareness of cancer and fearful perceptions of cancer in minority ethnic groups [7, 16, 23–26], which mirrors our finding that awareness of cancer curability was perceived to be low across South Asian faith communities. Within this study, informants representing the Sikh faith community described a ‘social stigma’ surrounding cancer which related to this low awareness. Previous studies have also identified ‘social stigma’ as a deterrent to breast and cervical cancer screening amongst mixed South Asian faith communities [23] and Somali Muslim women [27]. The experience and impact of ‘social stigma’ related to cancer would therefore benefit from further exploration. Finally we have identified potential difficulties associated with the sampling and storage of faeces in order to complete the gFOBt kit, including limitations on getting help from family members due to the personal nature of the test, which have similarly been reported within the majority (White European and African Caribbean) population [28–30].

There was agreement across faith communities in the preferred approaches to increase accessibility and awareness of CRC screening. In common with previous studies, we have identified an overwhelming preference for face-to-face and interactive approaches in order to provide information and raise awareness about the availability and purpose of CRC screening [7, 14]. We also identified a desire for the provision of information to take place ‘in language’ within community and/or faith settings [7, 16, 23]; and the potential value of ethnic community media to publicise the BCSP and gFOBt completion [7, 23]. A simplified test kit and the option to seek guidance from the GP or practice nurse were noted as further potential strategies to increase uptake.

Interviews with key informants generated culturally and contextually informed knowledge about how an invitation to the BSCP may be received within faith communities. We acknowledge the limitation that informants were expected to speak ‘on behalf of’ communities but may have given their personal views on CRC screening as well as reporting general cultural issues. Further, key informants are likely to have had a less detailed understanding of the BSCP invitation and gFOBt completion than participants with personal experience of being invited to screening.

We acknowledge that there is further diversity within each South Asian faith group that our sample was not able to include. Specifically, we were unable to recruit participants representing the Pakistani Muslim ethno-cultural group. Furthermore, the number of informants representing each faith community was small, and this may have meant that differences between communities in their likely responses to an offer of CRC screening remained unexamined. Although our study sample was limited to London, the shared ethnic and religious origins of South Asian communities across the UK are likely to mean our findings have wider relevance. Finally, we acknowledge that the language the interviews were conducted in (English) and the gender and ethnicity of the interviewer (white European and female) are likely to have impacted on the participant sample recruited.

## Conclusions

Our findings identify barriers to CRC screening uptake in the UK for many people within South Asian communities that persist despite being first identified over a decade ago [7]. Reasons for low uptake of CRC screening are predominantly shared across South Asian faith communities. However, indications of possible differences between communities require further research to determine specific cultural issues experienced in individual faith groups. Verbal and interactive approaches in the appropriate language for the target community should replace written messages mailed directly to individuals. Approaches should be delivered within community and faith settings, and be backed up with the use of local ethnic media using local language broadcasts. The use of community-specific lay workers and health fairs within faith settings in which multiple health problems are addressed are potentially valuable ways to engage South Asian communities. Given the role of children in mediating access to health for South Asian elders it may be beneficial to raise awareness of the BCSP across all age groups. Design and delivery of interventions may need to be tailored to the distinct needs of specific South Asian minority ethnic and faith groups.
